# Re-evaluating the predictive roles of metabolic complications and clinical outcome according to eGFR levels — a four-years prospective cohort study in Taiwan

**DOI:** 10.1186/1471-2369-14-92

**Published:** 2013-04-22

**Authors:** I-Wen Wu, Kuang-Hung Hsu, Chin-Chan Lee, Chiao-Yin Sun, Heng-Jung Hsu, Ming-Jui Hung, Mai-Szu Wu

**Affiliations:** 1 Department of Nephrology, Chang Gung Memorial Hospital, Keelung, Taiwan; 2 College of Medicine, Keelung, Taiwan; 3 Department of Health Care Management, Laboratory for Epidemiology, Healthy Aging Research Center, Chang Gung University, Taoyuan, Taiwan; 4 Department of Cardiology, Chang Gung Memorial Hospital, Keelung, Taiwan; 5 Division of Nephrology, Taipei Medical University Hospital, 252, Wu Hsiung Street, Taipei, 11031, Taiwan; 6 School of Medicine, Taipei Medical University, Taipei, Taiwan

**Keywords:** Anemia, Chronic kidney disease, Death, Hyperphosphatemia, Hypoalbuminemia, Metabolic complications, Renal progression

## Abstract

**Background:**

Metabolic complications are associated with clinical outcomes in patients with chronic kidney disease (CKD). These outcomes differ among patients according to the different stages of disease. The prevalence and association of type and number of metabolic complications with renal progression and death in patients having different eGFR levels has high clinical value, but this fact has been rarely evaluated in prospective studies.

**Methods:**

We prospectively followed a cohort of 1157 CKD patients from 2006 to death or until 2010, and evaluated the prevalence of CKD-related complications and their association with renal progression (defined as a decline in eGFR by > 50% from baseline, or end-stage renal disease requiring dialysis) and death in patients with eGFRs above and below 45 mL/min/1.73 m^2^ using Cox-proportional hazard models.

**Results:**

The estimated rate (per 100 patient-years) of renal progression and death were 11.9 and 4.9, respectively. The eGFR thresholds determined by ROC analysis with a sensitivity of 90% for any metabolic complication were 60.8 mL/min/1.73 m^2^ and 74.3 mL/min/1.73 m^2^ using the MDRD and CKD Epidemiology Collaboration equations, respectively. CKD-related complications associated with renal progression in patients having eGFR < 45 mL/min/1.73 m^2^ were hyperphosphatemia, anemia, microinflammation and hypoalbuminemia. Those CKD-related complications associated with death were hypoalbuminemia and hyperuricemia. Hypoalbuminemia predicted renal progression, and, hypoalbuminemia and microinflammation predicted death in patients with eGFR ≥ 45 mL/min/1.73 m^2^. The number of complications (≥ 3) independently predicted both endpoints in patients with eGFR < 45 mL/min/1.73 m^2^.

**Conclusions:**

Hypoalbuminemia was a unique and strong predictor of renal progression and all-cause mortality in CKD patients, independent of their demographic characteristics, traditional risk factors, renal function severity, the presence of cardiovascular disease and other metabolic abnormalities. Most other metabolic complications and the number of complications (≥3) were associated with the clinical outcomes of patients with eGFR < 45 mL/min/1.73 m^2^ rather than in those with higher eGFRs. The findings from the present study offer a novel insight into the association between metabolic complications and patient outcomes and may help to refine risk stratification according to disease stage.

## Background

The metabolic complications are associated with clinical outcomes in patients with chronic kidney disease (CKD). Patients with stage 3 CKD are more likely to die than undergo renal progression. In contrast, patients with stages 4 and 5 CKD who survive this life-threatening condition, are at greater risk of end-stage renal disease (ESRD) than death [1,2]. Numerous CKD-related metabolic complications develop undetected and asymptomatically at disease onset, before progression to renal failure and death. The associations renal progression and death with traditional risk factors and selected complications, such as anemia [3], calcium-phosphate imbalance [4], hyperkalemia [5], acidosis [6], hyperuricemia [7] and malnutrition–inflammation [8] have been extensively studied in patients with CKD. However, whether the type and number of CKD-related complications have similar impacts on clinical outcomes in patients with different estimated glomerular filtration rates (eGFRs) remains unclear.

The NKF/DOQI classification of CKD encompasses a wide range of patients within stage 3. The high estimates of the prevalence of this disease may partially represent an over diagnosis of kidney dysfunction due to misclassification of asymptomatic patients as having “pre-disease” or a “high-risk” condition when abnormal laboratory results arise. This may cause unnecessary concern among patients leading to the potential overuse of subspecialty resources. For this reason, a Report of KDIGO Controversies Conference proposed the subdivision of stage 3 into “3a” and “3b” strata at an eGFR of 45 mL/min/1.73 m^2^[9]. Elucidating the relationship among different metabolic complications with clinical outcomes in patients with eGFRs above and below 45 mL/min/1.73 m^2^ would be valuable. Given the high disease burden and the reversible nature of stage 3 CKD improving our knowledge of the factors associated with renal progression and death in patients with eGFRs above and below 45 mL/min/1.73 m^2^ is essential.

We conducted a prospective cohort study to (1) investigate the prevalence of CKD-related metabolic complications in patients with eGFR above and below 45 mL/min/1.73 m^2^, and, (2) evaluate the predictive role of these abnormalities on renal progression (defined as a decline in eGFR by >50% from baseline, or ESRD requiring dialysis) and all-cause death based on this eGFR cut-off.

## Methods

### Patient selection and study design

All participants were predialysis CKD patients who participated in the Multidisciplinary Predialysis Education program [10] at outpatient clinics held at the Department of Nephrology of Chang Gung Memorial Hospital at Keelung from May. Patients were followed up until April 15, 2010, or until they died, or were lost from the study. A total of 1157 patients aged 18–90 years without renal graft failure, or spontaneous improvement or progression of renal disease in the previous 3 months were enrolled in this study after informed consents were obtained. CKD was defined as having persistent proteinuria or a decreased eGFR of less than 60 mL/min/1.73 m^2^, determined by the abbreviated Modification of Diet in Renal Disease (MDRD) equation in 2 separate measurements within an interval of 3 months. Serum Cr was assessed by spectrophotometric analysis using a modified kinetic Jaffe reaction with standardization of the creatinine calibration to an isotope dilution mass spectrometry (IDMS) reference measurement procedure. This study was conducted in adherence with the *Declaration of Helsinki* and was approved by the Ethics Committee of the Institutional Review Board at the Chang Gung Memorial Hospital.

### Definitions

All clinical data and biological parameters were collected at baseline upon entry into the study. We defined CKD-related complications as follows: anemia, hemoglobin levels less than 13.5 g/dL in men or 12 g/dL in women [11] or the use of erythropoiesis-stimulating agents; hyperkalemia, K greater than 5 mEq/L or ion-exchange resin therapy; acidosis, CO_2_ less than 22 mmol/L or bicarbonate treatment; hypocalcemia, corrected Ca less than 8.5 mg/dL; hyperphosphatemia, P greater than 4.5 mg/dL or phosphate binder use; hyperparathyroidism, intact parathyroid hormone more than twice the upper limit of normal, corresponding to 70 pg/mL [12]; hyperuricemia, uric acid levels more than 8.0 mg/dL, or treatment with hypouricemic agents; and, hypoalbuminemia, serum albumin concentration less than 3.6 mg/dL. Obesity was evaluated according to the WHO classification as having a body mass index (BMI) of 30 kg/m^2^ or more. Renal function was checked during follow-up visits and at regular intervals according to the NKF/DOQI recommendations (stage 1 and 2 CKD patients were followed-up every 6 months; stage 3 or 4 CKD, every 3 months and stage 5 CKD patients, on a monthly basis). Renal progression was defined as a decline in eGFR by >50% from baseline or ESRD requiring dialysis. Mortality was carefully revised from the medical records. Patients or their relatives were telephoned when patients were lost to follow-up for longer than 3 months. Cases lost to follow-up in the subsequent visits were censored in the analysis. Deaths that occurred outside the hospital were verified by relatives during the telephone calls.

### Statistical methods

Descriptive statistics were expressed as the mean ± standard deviation, median (range), and/or frequency (percentage) where appropriate. All numerical variables were tested for normality using the Kolmogorov–Smirnov test. Data were log-transformed to approximate a normal distribution with right skewness. The Student’s *t* test or Mann–Whitney *U* test was applied to compare continuous variables between groups. Categorical data were analyzed using the chi-squared test. Cumulative renal survival and patient survival were estimated using the Kaplan–Meier method. Adjusted risk estimates for endpoints were calculated using univariate analyses followed by multivariate Cox proportional hazard regression analysis. Receiver operating characteristic (ROC) analysis was used to determine the optimal cutoff number of complications for outcome prediction. The presence of more than 3 metabolic complications discriminated between patients with and without renal progression (sensitivity, 81% and specificity, 67%) and death (sensitivity, 76% and specificity, 61%). The power of the contribution of each variable to the model fit was estimated by determining the percent reduction of the pseudo-R^2^ value resulting from the sequential omission of the maximum likelihood-ratio of each variable from the complete model [13]. The assumption of proportionality was checked graphically using complementary log-log plots and was found to be acceptable for the risk factors of interest. All statistical tests were 2-tailed, and a *p*-value less than 0.05 was considered statistically significant. Data were analyzed using the SPSS 17.0 software for Windows XP (SPSS Inc., Chicago, IL).

## Results

**Figure 1:**
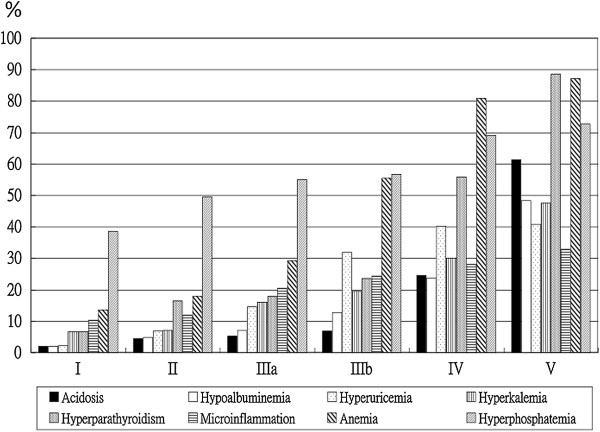
Prevalence of metabolic complications according to stages of chronic kidney disease.

The mean age of the study patients was 66.8 ± 13 years, and 640 (55.3%) patients were men. The mean eGFR was 32.0 ± 26.3 mL/min/1.73 m^2^. Diabetes mellitus (DM) was the leading cause of renal disease (43.5%). Table 1 summarizes the baseline characteristics of the study population. Of the 1157 patients who were enrolled in the study, 1068 (92.3%) patients presented with at least one metabolic complication. The eGFR thresholds determined by ROC analysis with a sensitivity of 90% for any metabolic complication were 60.8 and 74.3 mL/min/1.73 m^2^, using MDRD and CKD Epidemiology Collaboration equations, respectively (Table 2). Figure 1 summarizes the prevalence of metabolic complications according to the stages of CKD.

**Table 1:** Demographic characteristics stratified by CKD stages (n = 1157)

	**Stage 1**	**Stage 2**	**Stage 3a**	**Stage 3b**	**Stage 4**	**Stage 5**
	**(n = 44)**	**(n = 102)**	**(n =96)**	**(n = 195)**	**(n = 389)**	**(n = 331)**
Age, years	55.6 ± 13.5*	59.5 ± 14.1*	65.8 ± 13.8*	68.2 ± 12.9*	69.3 ± 12.8*	67.4 ± 12.3
Male, No. (%)	11 (25%)	57 (55.9%)	68 (70.8%)	135 (69.2%)	216 (55.5%)	156 (47.1%)
Diabetes, No. (%)	17 (38.6%)	25 (24.5%)	27 (28.1%)	86 (44.1%)	212 (54.6%)	193 (58.3%)
Hypertension, No. (%)	23 (52.3%)	44 (43.1%)	66 (68.8%)	149 (76.4%)	312 (80.4%)	272 (82.2%)
Primary cause						
Diabetes, No. (%)	16 (36.4%)	18 (17.8%)	29 (29.2%)	77 (39.6%)	193 (49.6%)	175 (52.9%)
Hypertension, No. (%)	4 (9.1%)	14 (13.9%)	14 (14.6%)	12 (6%)	27 (6.9%)	8 (2.4%)
CGN, No. (%)	3 (6.8%)	4 (4%)	2 (2.2%)	17 (8.8%)	46 (11.7%)	64 (19.2%)
Gout, No. (%)	2 (4.5%)	21 (20.8%)	23 (22.5%)	37 (18.7%)	48 (12.3%)	17 (5.1%)
Obstructive uropathy, No. (%)	0 (0%)	0 (0%)	0 (0%)	3 (1.6%)	5 (1.4%)	12 (3.7%)
ADPKD, No. (%)	1 (2.3%)	1 (1%)	0 (0%)	5 (2.7%)	7 (1.7%)	3 (1%)
Unknown/other, No. (%)	18 (40.9%)	43 (42.6%)	30 (31.5%)	43 (22.5%)	63 (16.3%)	52 (15.8%)
Body mass index, kg/m2	27.1 ± 5.9*	25.5 ± 3.6*	26.0 ± 4.8*	25.7 ± 3.6*	25.8 ± 4.1 *	24.8 ± 4.0
Systolic BP, mmHg	131 ± 18	131 ± 17	135 ± 16	134 ± 18	137 ± 20	140 ± 21
Diastolic BP, mmHg	75 ± 9	75 ± 9	77 ± 10	73 ± 11	71 ± 11	72 ± 10
Laboratory						
eGFR, mL/min per 1.73 m2 (MDRD)	112.8 ± 30.8*	72.6 ± 8.4*	51.8 ± 4.2*	36.2 ± 3.8*	22.1 ± 4.1*	9.4 ± 3.2
eGFR, ml/min per 1.73 m2 (EPI)	115.2 ± 32.4*	76.8 ± 10.2*	62.5 ± 11.9*	41.7 ± 8.5*	23.4 ± 6.9*	9.7 ± 4.0
eGFR, mL/min per 1.73 m2 (CG)	119 ± 35.8*	81.2 ± 18.7*	65.5 ± 41.3*	26.7 ± 8.7*	12.3 ± 4.3*	12.2 ± 4.3*
BUN, mg/dL	14 ± 5*	15 ± 5*	19 ± 7*	25 ± 10*	28 ± 15*	67 ± 29
Serum creatinine, mg/dL	0.6 ± 0.1*	0.9 ± 0.1*	1.1 ± 0.1*	1.5 ± 0.1*	2.4 ± 0.4*	5.5 ± 2.2
Hemoglobin, g/dL	13.7 ± 1.6*	14.1 ± 1.7*	13.9 ± 1.9*	12.7 ± 2.1*	11.3 ± 2.0*	9.6 ± 1.6
Serum sodium, mEq/L	140 ± 2.4	141 ± 2.1	141 ± 2.6	139 ± 3.4	138 ± 7.7	137 ± 10
Serum potassium, mEq/L	4.1 ± 0.4*	4.2 ± 0.6*	4.4 ± 0.5*	4.3 ± 0.6*	4.5 ± 0.6*	4.7 ± 0.8
Serum calcium, mg/dL	9.3 ± 0.4	9.3 ± 0.3	9.4 ± 0.5	9.3 ± 0.5	9.2 ± 0.6	8.6 ± 0.7
Serum phosphate, mg/dL	4.1 ± 1	4.4 ± 0.9	4.4 ± 1.6	4.7 ± 1.7	4.7 ± 1.7	4.9 ± 1.3
Serum chloride, mEq/L	104 ± 3	103 ± 9	104 ± 3	104 ± 9	105 ± 8	103 ± 17
Serum bicarbonate, mmol/L	26.6 ± 2.1*	27.2 ± 2.9*	27.5 ± 2.4*	26.4 ± 2.9*	24.6 ± 3.4*	20.8 ± 4.5
Serum uric acid, mg/dL	4.8 ± 1.4*	4.9 ± 1.6*	5.5 ± 2.4*	6.7 ± 2.3*	7.7 ± 2.0*	7.8 ± 1.9
Serum albumin, g/dL	4.2 ± 0.4*	4.2 ± 0.3*	4.1 ± 0.4*	3.9 ± 0.6*	3.7 ± 0.6*	3.5 ± 0.6
Cholesterol, mg/dL	213 ± 53*	206 ± 41*	199 ± 44*	199 ± 45*	195 ± 47*	191 ± 53
iPTH, pmol/L	43.2 (13.1, 87.3)*	48.4 (13.8, 135)*	53.2 (3.9, 169)*	52.9 (1, 158)*	98.3 (4, 400)*	196 (23.5, 1632)
hs-CRP, mg/L	1.5 (0.2, 21.3)*	1.0 (0.2, 56.4)*	1.66 (0.2, 16.2)*	2.0 (0.2, 119.1)*	2.6 (0.2, 63.3)*	3.5(0.2, 187)
Microalbuminuria, mg/day	225 ± 46*	291 ± 25*	391 ± 12*	949 ± 21*	739 ± 10*	780 ± 61
Cardiovascular disease, No. (%)	2 (4.5%)	6 (5.9%)	9 (9.4%)	43 (22.1%)	87 (22.4%)	78 (23.6%)
Use of ACEI/ARB, No. (%)	3 (6.8%)	6 (5.9%)	11 (11.2%)	82 (42.3%)	226 (58.2%)	219 (66.3%)

The values are expressed as means (SD) or median (Min, Max). CGN, chronic glomerulonephritis; ADPKD; autosomal dominant.

polycystic disease; BP; blood pressure; eGFR, estimated glomerular filtration rate; MDRD, Modification of Diet in Renal Disease; EPI, CKD epidemiology.

Collaboration; CG, Cockcroft-Gault; BUN; blood urea nitrogen; iPTH, intact parathyroid hormone; hs-CRP, high-sensitivity C-reactive protein;

ACEI, angiotensin-converting enzyme; ARB, angiotensin II receptor blocker; *p < 0.05 vs. stage V.

**Table 2:** eGFR thresholds associated with metabolic complications with a sensitivity of 90%

**Complications**	**eGFR, mL/min/1.73 m2**	**Specificity**	**eGFR, mL/min/1.73 m2**	**Specificity**
	**(Abbreviated MDRD)**	**%**	**(CKD-EPI)**	**%**
Hyperphosphatemia	55.3	28	86.3	64
Hyperparathyroidism	35.8	50	39.9	53
Anemia	40.5	58	44.2	64
Hyperuricemia	39.1	35	45.6	33
Hyperkalemia	41	31	40.1	36
Hypoalbuminemia	32.9	45	35.4	45
Acidosis	29.8	59	27.8	59
Microinflammation	43.3	26	57.8	21

eGFR, estimated glomerular filtration rate; MDRD, Modification of Diet in Renal Disease; CKD-EPI, Chronic Kidney Disease Epidemiology Collaboration.

After a mean follow-up of 24.5 ± 12.5 months (2368.6 patient-years), 283 (24.5%) patients (11.9 per 100 patient-years) had renal progression and 118 (10.2%) patients (4.9 per 100 patient-years) had died. Causes of death included cardiovascular diseases (28 patients), infection (50 patients), gastrointestinal bleeding (4 patients), malignancies (3 patients), respiratory disorders (2 patients), hepatic diseases (2 patients), and other diseases (29 patients). The incidences of renal progression and death were 1.8 and 2.0 per 100 patient-years, respectively, for patients with eGFR ≥ 45 mL/min/1.73 m^2^; and 14.7 and 5.8 per 100 patient-years, respectively, for patients with eGFR below 45 mL/min/1.73 m^2^.

Table 3 lists the univariate hazard ratios for renal progression and all-cause mortality according to the eGFR levels. In patients with eGFR < 45 mL/min/1.73 m^2^, number of metabolic complications (more than 3, HR: 2.18; 95% CI: 1.48–3.22; *p* < 0.001) was an independent risk factor for renal progression after adjustment for patient demographic characteristics (age, gender, DM and CKD stage, model 1, Table 4). Independent associations and the hierarchical analyses of diverse metabolic complications with renal progression are reported in model 2 of Table 4. These factors included hyperphosphatemia (HR: 2.09; 95% CI: 1.57–2.80; *p* < 0.001), anemia (HR: 2.64; 95% CI: 1.38–5.01; *p* = 0.003), hypoalbuminemia (HR: 2.58; 95% CI: 2.04–3.57; *p* < 0.001) and microinflammation (HR: 1.36; 95% CI: 1.02–1.80; *p* = 0.038, Table 4). For patients with eGFR ≥ 45 mL/min per 1.73 m^2^, only hypoalbuminemia (HR: 3.25; 95% CI: 1.73–4.61; *p* <0.001) was independently associated with renal progression (Table 4).

**Table 3:** Univariate hazard ratios for renal progression and all-cause mortality according to eGFR levels

	**Renal progression**				**All-cause mortality**			
	**eGFR ≥45 mL/min/1.73 m2**		**eGFR <45 mL/min/1.73 m2**		**eGFR ≥45 mL/min/1.73 m2**		**eGFR <45 mL/min/1.73 m2**	
Variable	HR (95% CI)	*p*	HR (95% CI)	*p*	HR (95% CI)	*p*	HR (95% CI)	*p*
Age	1.04 (0.93 - 1.09)	0.18	0.98 (0.97 - 0.99)	<0.001	1.13 (1.05 - 1.22)	0.002	1.06 (1.04 - 1.08)	<0.001
Male gender	1.05 (0.28 - 3.93)	0.948	0.75 (0.59 - 0.94)	0.015	0.57 (0.16 - 2.02)	0.38	0.75 (0.52 - 1.09)	0.139
Diabetes (yes vs. no)	4.89 (1.22 - 9.61)	0.025	1.88 (1.47 - 2.42)	<0.001	1.08 (0.28 - 4.18)	0.91	1.36 (0.93 - 1.99)	0.118
Hypertension (yes vs. no)	3.34 (0.62 - 18.14)	0.274	1.34 (0.87 - 2.08)	0.189	3.41 (0.10 - 11.34)	0.234	0.82 (0.46 - 1.47)	0.503
Hypercholesterolemia (yes vs. no)	0.61 (0.16 - 2.29)	0.464	1.06 (0.83 - 1.35)	0.641	0.55 (0.15 - 1.97)	0.361	0.79 (0.55 - 1.16)	0.234
Smoking (yes vs. no)	6.15 (1.27 - 9.82)	0.024	0.95 (0.74 - 1.22)	0.679	2.67 (0.75 - 9.51)	0.13	0.95 (0.64 - 1.41)	0.789
Obesity (yes vs. no)	0.89 (0.11 - 6.98)	0.858	1.17 (0.84 - 1.62)	0.354	1.46 (0.31 - 6.87)	0.635	0.65 (0.34 - 1.25)	0.195
Hyperphosphatemia (yes vs. no)	0.13 (0.02 - 1.06)	0.057	4.27 (3.29 - 5.53)	<0.001	0.31 (0.07 - 1.49)	0.143	1.39 (0.95 - 2.05)	0.088
Hyperparathyroidism (yes vs. no)	0.69 (0.08 - 5.58)	0.725	2.69 (2.31 - 3.57)	<0.001	2.12 (0.27 - 16.75)	0.477	2.01 (1.32 - 3.04)	0.001
Anemia (yes vs. no)	1.15 (0.37 - 6.22)	0.567	11.9 (5.33 - 26.8)	<0.001	2.22 (0.62 - 7.71)	0.225	2.63 (1.37 - 5.05)	0.004
Hyperuricemia (yes vs. no)	0.04 (0.01 - 1.82)	0.563	0.87 (0.67 - 1.11)	0.266	2.45 (0.52 - 11.55)	0.258	1.99 (1.37 - 2.92)	<0.001
Hyperkalemia (yes vs. no)	3.78 (0.47 - 30.7)	0.214	1.69 (1.31 - 2.19)	<0.001	3.16 (0.39 - 25.05)	0.276	1.11 (0.71 - 1.74)	0.649
Hypoalbuminemia (yes vs. no)	5.02 (1.01 - 7.42)	0.004	4.02 (3.16 - 5.12)	<0.001	4.79 (1.38 - 6.51)	0.013	3.56 (2.43 - 5.22)	<0.001
Acidosis (yes vs. no)	0.49 (0.01 - 1.01)	0.756	3.35 (2.64 - 4.26)	<0.001	5.18 (0.64 - 42.12)	0.124	1.72 (1.17 - 2.53)	0.006
Microinflammation (yes vs. no)	2.43 (0.49 - 12.1)	0.276	1.33 (1.03 - 1.71)	0.03	4.82 (1.36 - 7.10)	0.015	1.35 (0.89 - 2.03)	0.148
CVD (yes vs. no)	2.81 (2.61 - 18.2)	0.004	4.56 (2.25 - 9.27)	<0.001	0.05 (0.01 - 20.33)	0.695	2.22 (1.35 - 3.64)	0.002
Metabolic complications (≥3 vs. <3)	1.54 (0.19 - 12.5)	0.688	5.62 (3.80 - 8.32)	<0.001	1.25 (0.16 - 9.48)	0.835	4.35 (2.47 - 7.63)	<0.001

HR, hazard ratios; CI, confidence interval; CKD, chronic kidney disease; CVD, cardiovascular disease.

**Table 4:** Multivariate Cox regression analysis of renal progression with the estimated contribution of each covariates to the prediction

	**HR**	***p***	**R2 reduction**
	**(95% CI)**		**(%)**
**eGFR ≥ 45 ml/min/1.73 m2**			
Diabetes (yes vs. no)	1.91 (1.18 - 2.11)	0.035	1.11
Hypoalbuminemia (yes vs. no)	3.25 (1.73 - 4.61)	<0.001	6.91
CVD (yes vs. no)	2.81 (2.61 - 4.82)	0.004	2.39
**eGFR < 45 ml/min/1.73 m2**			
**Model 1**			
Age	0.98 (0.97 - 0.99)	<0.001	0.70
Gender (male vs. female)	0.93 (0.74 - 1.18)	0.059	0.10
Diabetes (yes vs. no)	1.71 (1.42 - 2.34)	<0.001	1.29
CKD stage	2.67 (2.19 - 3.26)	<0.001	7.67
Metabolic complications (≥3 vs. <3)	2.18 (1.48 - 3.22)	<0.001	0.51
**Model 2**			
Age	0.98 (0.97 - 0.99)	<0.001	0.74
Gender (male vs. female)	1.05 (0.81 - 1.37)	0.704	<0.001
CKD stage	2.30 (1.87 - 2.83)	<0.001	5.68
CVD (yes vs. no)	3.29 (1.56 - 6.99)	0.002	0.29
Hyperphosphatemia (yes vs. no)	2.09 (1.57 - 2.80)	<0.001	1.26
Hyperparathyroidism (yes vs. no)	0.92 (0.64- 1.31)	0.633	<0.001
Anemia (yes vs. no)	2.64 (1.38 - 5.01)	0.003	0.41
Hyperuricemia (yes vs. no)	1.03 (0.78 - 1.35)	0.857	<0.001
Hypoalbuminemia (yes vs. no)	2.58 (2.04 - 3.57)	<0.001	4.54
Acidosis (yes vs. no)	1.24 (0.93 - 1.67)	0.15	<0.001
Microinflammation (yes vs. no)	1.36 (1.02 - 1.80)	0.038	0.15

HR, hazard ratios; CI, confidence interval; CKD, chronic kidney disease; CVD, cardiovascular disease.

Model 1: adjusted for patient demographic characteristics, CKD stage and number of complications.

Model 2: hierarchical analyses of diverse metabolic complications adjusted for age, gender and CKD stage.

**Figure 2:**
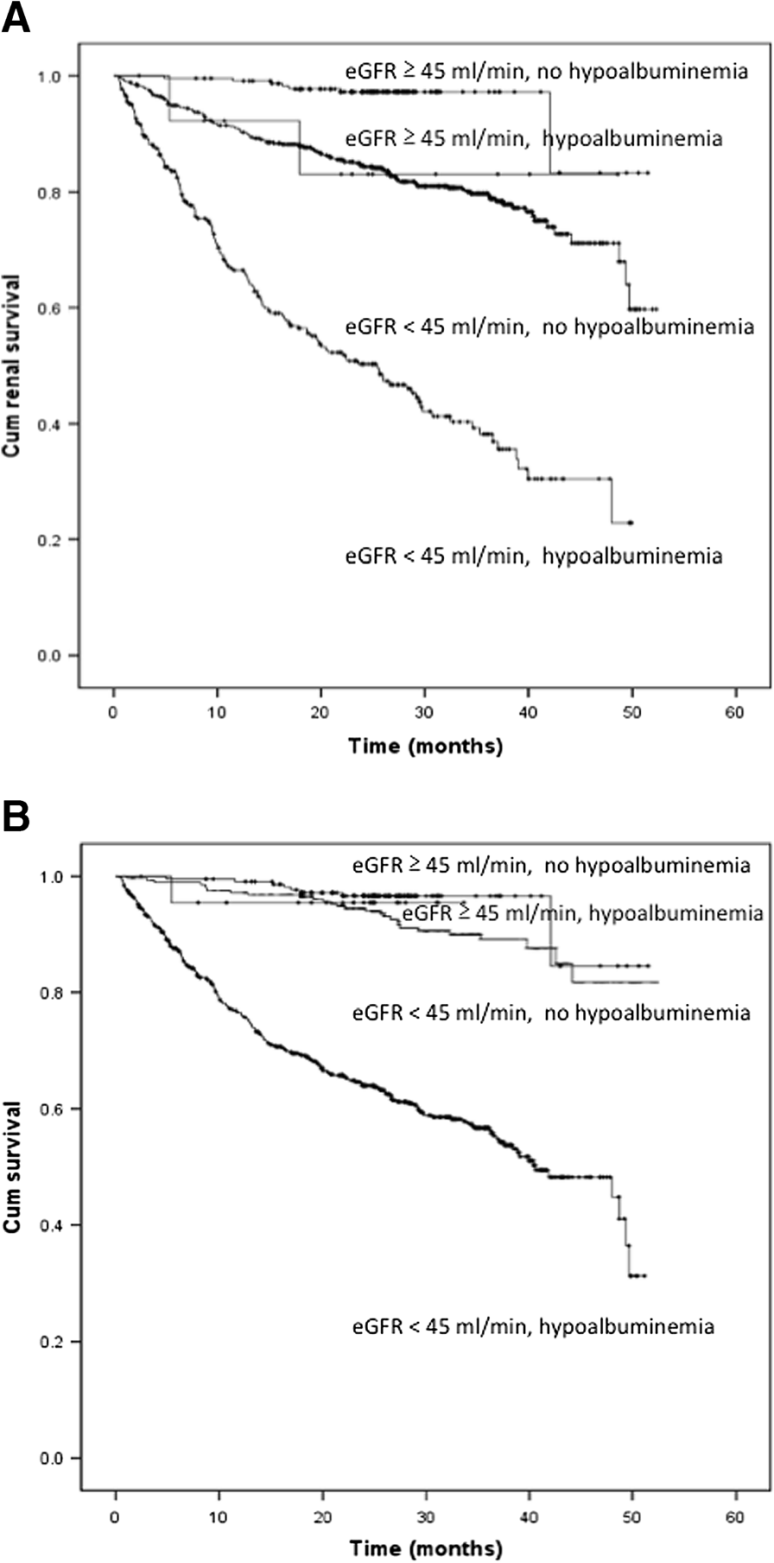
**Cumulative renal survival (A) and overall survival (B) based on hypoalbuminemia in patients with eGFR above or below 45 mL/min per 1.73 m**^**2 **^**(log-rank test, *p *< 0.001).**

**Figure 3:**
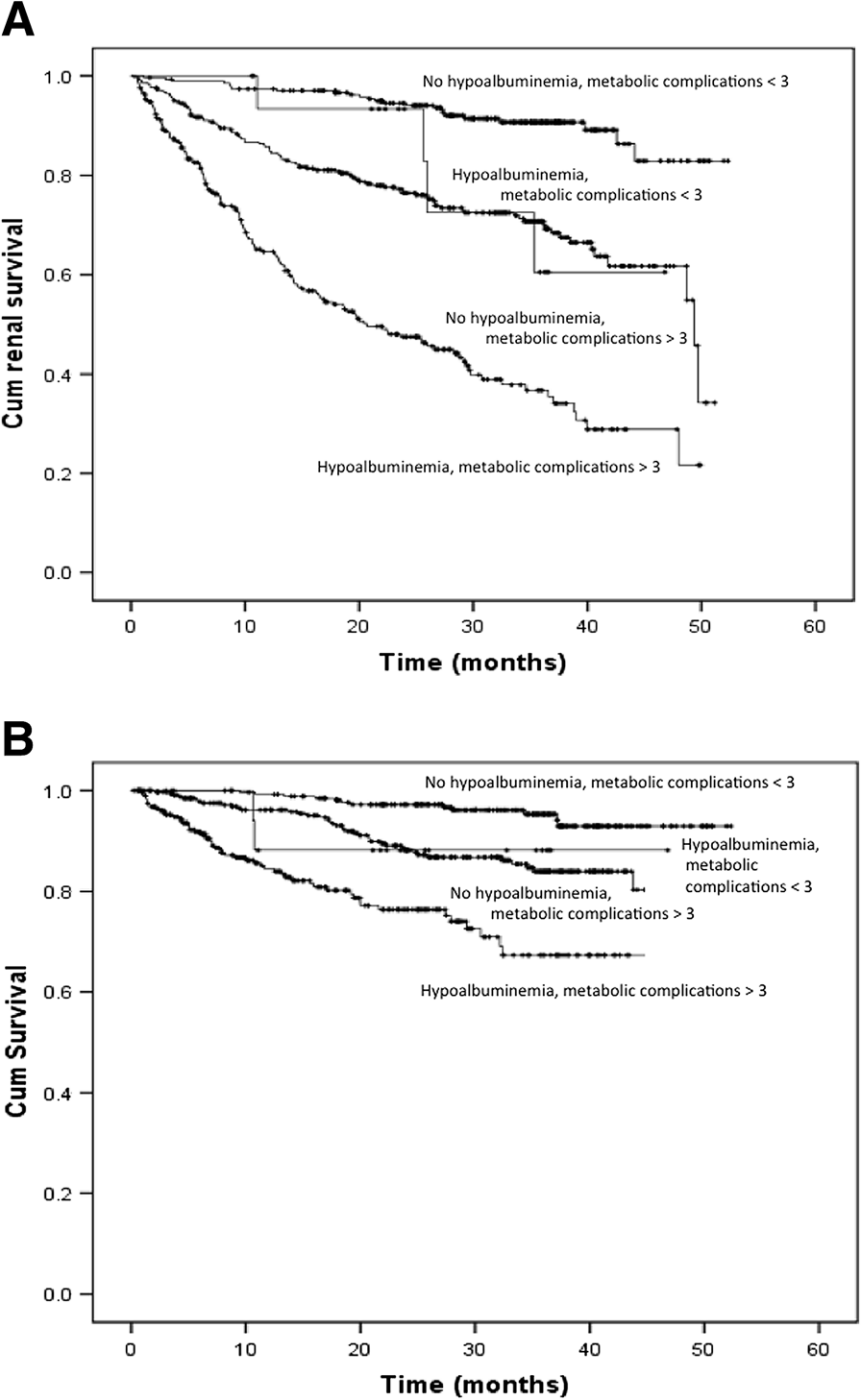
**Cumulative renal (A) and overall survival (B) based on hypoalbuminemia and presence of more than 3 metabolic complications in patients with eGFR below 45 mL/min per 1.73 m**^**2 **^**(log-rank test, *p*** **< 0.001).**

The presence of ≥3 metabolic complications (HR: 3.71; 95% CI: 2.35–5.87; *p* < 0.001, model 1), hyperuricemia (HR: 2.09; 95% CI: 1.39–3.13; *p* < 0.001) and hypoalbuminemia (HR: 2.39; 95% CI: 1.54–3.69; *p* < 0.001, model 2) independently predicted death in patients with eGFR < 45 mL/min/1.73 m^2^ (Table 5). Hypoalbuminemia (HR: 5.87; 95% CI: 1.22–8.21; *p* = 0.001) and microinflammation (HR: 3.31; 95% CI: 1.61–7.82; *p* = 0.027) independently predicted death in patients with higher eGFR (Table 5). The presence of ≥3 metabolic complications and hypoalbuminemia had the greatest contribution to the prediction of both renal progression and death in patients with eGFR < 45mL/min/1.73 m^2^, as determined by percent reduction of pseudo- R^2^ values. Figure 2 shows the cumulative renal survival and overall survival according to eGFRs and the presence of hypoalbuminemia. Cumulative renal survival and overall survival, according to the number of complications and hypoalbuminemia in patients with eGFR < 45 mL/min/1.73 m^2^ are illustrated in Figure 3.

**Table 5:** Multivariate Cox regression analysis of all-cause mortality with estimated contribution of each covariate to the prediction

	**HR**	***p***	**R2 reduction**
	**(95% CI)**		**(%)**
**eGFR ≥ 45 ml/min/1.73 m2**			
Age	1.11 (1.03 - 1.19)	0.006	16.13
Hypoalbuminemia (yes vs. no)	5.87 (1.22- 8.21)	0.001	5.61
Microinflammation (yes vs. no)	3.31 (1.61 - 7.82)	0.027	4.45
**eGFR < 45 ml/min/1.73 m2**			
**Model 1**			
Age	1.06 (1.04 - 1.08)	<0.001	3.70
Gender (male vs. female)	0.84 (0.59 - 1.21)	0.36	<0.001
Diabetes (yes vs. no)	1.36 (094–1.97)	0.126	<0.001
CKD stage	1.13 (0.91 - 1.41)	0.279	<0.001
Metabolic complications (≥3 vs. <3)	3.71 (2.35 - 5.87)	<0.001	2.60
**Model 2**			
Age	1.07 (1.47 - 1.09)	<0.001	5.00
Gender (male vs. female)	0.66 (0.45 - 0.10)	0.061	0.14
CKD stage	1.37 (1.07 - 1.75)	0.012	0.68
CVD (yes vs. no)	1.86 (1.09 - 3.19)	0.024	0.39
Hyperphosphatemia (yes vs. no)	0.98 (6.23 - 1.55)	0.939	<0.001
Hyperparathyroidism (yes vs. no)	1.36 (0.97 - 2.42)	0.223	<0.001
Anemia (yes vs. no)	1.10 (0.61 - 2.00)	0.924	<0.001
Hyperuricemia (yes vs. no)	2.09 (1.39 - 3.13)	<0.001	1.37
Hypoalbuminemia (yes vs. no)	2.39 (1.54 - 3.69)	<0.001	2.11
Acidosis (yes vs. no)	1.37 (0.87 - 2.17)	0.173	<0.001
Microinflammation (yes vs. no)	1.46 (0.95 - 2.24)	0.083	<0.001

HR, hazard ratios; CI, confidence interval; CKD, chronic kidney disease; CVD, cardiovascular disease.

Model 1: adjusted for patient demographic characteristics, CKD stage and number of complications.

Model 2: hierarchical analyses of diverse metabolic complications adjusted for age, gender and CKD stage.

## Discussion

The KDIGO Controversies Conference on the definition and classification of CKD proposed the subdivision of stage 3 of the KDOQI classification into two stages (above and below 45 ml/min per 1.73 m^2^) due to the steep rise in risks (all-cause and cardiovascular mortality, kidney failure, acute kidney injury and progressive CKD) associated with lower eGFR [9]. In the present prospective study we evaluated the associations of different types and numbers of CKD-related metabolic complications with renal progression and all-cause mortality in patients with eGFRs above and below 45 mL/min/1.73 m^2^. We found that hypoalbuminemia was a unique and common predictor of renal progression and death in both groups of patients, independent of the patients’ demographic characteristics, traditional risk factors, severity of renal dysfunction and other metabolic abnormalities. The number of metabolic complications (≥ 3) was an independent factor associated with renal progression and death in patients with eGFR < 45 mL/min/1.73 m^2^. Most CKD-related metabolic complications were associated with renal progression and death in patients with eGFR < 45 mL/min/1.73 m^2^ rather than in those patients with higher eGFR. Metabolic complications associated with renal progression in patients with eGFR < 45 mL/min were hyperphosphatemia, anemia, microinflammation and hypoalbuminemia. Those associated with death were hypoalbuminemia and hyperuricemia. Whereas, in patients with eGFR ≥ 45 mL/min1.73 m^2^, hypoalbuminemia predicted renal progression, and hypoalbuminemia and microinflammation predicted death. Prospective studies evaluating the associations of all CKD-related metabolic complications with outcomes in patients with eGFRs above and below 45 mL/min/1.73 m^2^ have been rarely reported. Our findings may offer novel insight into the associations between metabolic complications and patient outcomes based on the stage of CKD.

Hypoalbuminemia is the main feature of the malnutrition-inflammation-atherosclerosis complex and it is probably associated with CKD as well as many other conditions. However, the significant association between hypoalbuminemia and patient outcomes noted in this study despite adjustments for all these conditions again highlights the importance of this risk factor in patient prognosis (models 1 and 2 of Tables 4 and 5). Sarcopenia and a lower BMI were also considered as possible causes of hypoalbuminemia. In the current study, the prevalence rates of sarcopenia, which manifests itself clinically in the presence of fractures (defined by an ICD-9 coding of 8000-8290), and underweight status (defined according to the WHO classification, as a BMI <18 kg/m^2^) were 2.2% and 2.1%, respectively. The contribution of sarcopenia and underweight status as possible intermediates or confounders of hypoalbuminemia may be small because of the low prevalence of these 2 conditions in our study population.

The strong independent association of hypoalbuminemia with renal progression and death in CKD patients, stronger than all other CKD-related metabolic complications, is important clinically. Findings from the Atherosclerosis Risk in Community Study revealed that serum albumin levels were inversely correlated with the risk of decline of renal function, suggesting a unique role for hypoalbuminemia as an antecedent pathway for CKD [8]. Like previous investigations, our study revealed a significant association between low serum albumin levels and mortality, independent of inflammation markers, such as C-reactive protein [14]. The NKF/DOQI guidelines suggested monthly measurements of serum albumin levels in maintenance dialysis patients [15]. However, despite abundant evidence, clinical guidelines are required to standardize the timing for screening serum albumin in predialysis CKD patients. Further, large-scale studies are needed to investigate the clinical improvement of patients who have had their hypoalbuminemia corrected.

The reasons for the apparent insignificance of metabolic complications in predicting outcomes, despite the high prevalence of certain complications (e.g., hyperphosphatemia and anemia) in patients with eGFR ≥ 45 mL/min/1.73 m^2^ should be investigated. One possible explanation is that metabolic complications may reflect both the endocrine and excretory functions of the kidneys and may not be sufficient to cause organ dysregulation. The presence of comorbidities and harmful systemic conditions may be more significant than the subtle dysfunction of a simple organ. These divergent findings clearly indicate the heterogeneous clinical course and dissimilar biological behaviors of patients with eGFR below or above 45 mL/min/1.73 m^2^. Further, these findings provide evidence that will help refine risk stratification and to establish the effective allocation of medical resources in an effort to alleviate the significant burden of CKD. In patients whose eGFRs are above 45 mL/min/1.73 m^2^, more attention should be paid to the treatment of comorbidities rather than CKD-related complications, while, vigorous treatment of CKD-related complications should be emphasized for late-stage patients.

In the present study, most of the cutoff points chosen to define metabolic complications were based on current guidelines. However, comparison of our findings with those from others groups may be difficult because the thresholds may have changed due to variations in guidelines over time and the different definitions of renal progression used in studies [16,17]. Notably, the time of onset of CKD-related complications in our patients was quite different from that reported in other studies [16-18]. For example, the eGFR threshold for the development of hyperphosphatemia in our patients was approximately 10 mL/min per 1.73 m^2^ higher than that in Caucasian patients [17]. The unadjusted prevalence rates of hyperphosphatemia were approximately 5.5-fold and 3-fold higher in our stages 1-3 and stages 4 and 5 patients, respectively, than the prevalence rates of hyperphosphatemia in participants from the same eGFR strata in the National Health and Nutrition Examination Survey [18]. Genetic backgrounds can influence the clinical features and outcomes in CKD patients of different ethnic origins [19]. For example, Africans are more likely to develop anemia, hyperphosphatemia and hyperuricemia and have a higher eGFR threshold for its development than Caucasians [20], while Oriental Asian patients generally experience more metabolic complications such as anemia, hypocalcemia, and hyperparathyroidism than Caucasians at all eGFRs levels [16]. The exact reason for these discrepancies in the development thresholds for these different cohorts is unclear. However, differences among the study populations, comorbidities, dietary habits, ethnicity-related PTH resistances, vitamin D metabolism, fibroblast growth factor-23 levels, education perceptions and compliance may partly influence this phenomenon. Paradoxically, Asian patients have greater survival advantages, despite these metabolic abnormalities [21]. In this study, we found that the most metabolic abnormalities are more likely to be associated with adverse outcomes only in patients with eGFR < 45 ml/min per 1.73 m^2^. Further large-scale clinical trials should be conducted to clarify the significance of metabolic complications on clinical outcomes in Asian CKD patients.

The strengths of our study include the study design, which was a large prospective cohort of patients with diverse renal functions, the collection of complete measurements of common traditional risk factors and CKD-related metabolic complications, and follow-up using a standardized program, which limited the number of noncompliant participants and facilitated the accurate recording of patients’ renal functions and outcomes. In the clinical setting, the use of a composite of a decline in eGFR by > 50% and the presence of ESRD requiring dialysis, to define renal progression, may have facilitated the timely detection of changes in renal function in patients with early-stage CKD. However, there were several limitations to this analysis, including the interrogation of a homogeneous Asian population from a single center and the lack of analysis of the time-dependent variations. The findings of the present study should not be interpreted in causal terms. The analysis of time-dependent variations in individual complications has been extensively described in other studies [22-25]. Reference to baseline complications in the assessment of patient outcomes may provide useful information to help clinicians make prognoses and to facilitate decision-making regarding CKD complications and outcomes when it is initially detected. We applied the most commonly used methods to estimate eGFR (i.e., the MDRD and CKD-Epidemiology Collaboration equations) rather than the gold-standard method (iothalamate GFR). The later method does not appear to be superior to equation-based estimations of eGFR in the explanation of CKD-related complications, except anemia [26].

## Conclusions

In this prospective study, we have demonstrated that hypoalbuminemia is a unique and strong predictor of renal progression and all-cause mortality in CKD patients, independent of demographic characteristics, traditional risk factors, severity of renal dysfunction, the present of cardiovascular disease, and other metabolic abnormalities. Most of other metabolic complications and the number of complications (more than 3) were associated with the clinical outcomes of patients who had eGFR < 45 mL/min/1.73 m^2^ rather than in those with higher eGFRs. The findings from this study offer a novel insight into the association between metabolic complications and clinical outcomes, and may help to refine risk stratification based on the stage of CKD.

## Competing interest

The authors declare that they have no competing interests.

## Authors’ contributions

I-WW participated in data collection, statistical analysis and manuscript preparation; KHH participated in statistical analysis; CCL helped to data collection; CYS helped to data collection; HJH helped to data collection; MJH participated in data interpretation; MSW participated in study design, coordination and helped to draft the manuscript. All authors read and approved the final manuscript.

## Pre-publication history

The pre-publication history for this paper can be accessed here:

http://www.biomedcentral.com/1471-2369/14/92/prepub
